# Not All that Strictures Is IBD: Gastric Heterotopia Leading to Perforation and Small Bowel Obstruction Mimicking IBD

**DOI:** 10.1097/PG9.0000000000000106

**Published:** 2022-02-04

**Authors:** Amariel Ralbovsky, Troy Reyna, Mustafa Kabeer, Ali Nael, Christine Yang

**Affiliations:** From the Department of Pediatrics, University of California Irvine, Children’s Hospital Orange County, Orange, CA.

**Keywords:** inflammatory bowel disease, heterotopic gastric mucosa, confocal laser microscopy, stricture, ectopic

## Abstract

A 14-year-old male presented with worsening chronic intermittent abdominal pain, mild anemia, positive fecal occult blood test, and elevated calprotectin. Computerized tomography and magnetic resonance imaging showed ileal dilation with mucosal enhancement and inflammatory changes suspicious for inflammatory bowel disease (IBD). Prominent mucosal folds were suggestive of gastric heterotopia, but Meckel’s scan was negative. Upper endoscopy, colonoscopy, and double balloon enteroscopy were grossly and microscopically normal. Laparotomy revealed 17 cm of a dense, inflamed, stenotic segment of ileum. The strictured ileum had perforated and had been concealed by an adjacent loop of small bowel, ultimately producing an obstructive IBD-like picture, but was found to be histologically consistent with gastric oxyntic mucosa. This case illustrates the challenges of diagnosing and treating heterotopic gastric mucosa, and the importance of considering diagnoses other than IBD when evaluating stricturing disease of the small bowel.

## INTRODUCTION

Heterotopic gastric mucosa (HGM) is an abnormal histological finding in which oxyntic gastric tissue, identifiable by an abundance of parietal and chief cells, is found elsewhere in the gastrointestinal tract (1–3). This tissue has no anatomical or vascular connection to the adjacent tissue. In the pediatric population, HGM is usually found to be heterotopic and congenital in origin, but can be metaplastic secondary to underlying inflammation or pathology (2). HGM produces gastric acid, causing inflammatory changes, injury, and possible perforation of adjacent tissue (1, 4–6). We report a case of HGM presenting with symptoms and imaging consistent with stricture, thought initially to be secondary to inflammatory bowel disease (IBD), and discuss the importance of keeping a broad differential in this case with negative testing and unusual symptomatology. Consent for publication was obtained from the family.

## CASE REPORT

**FIGURE 1.**
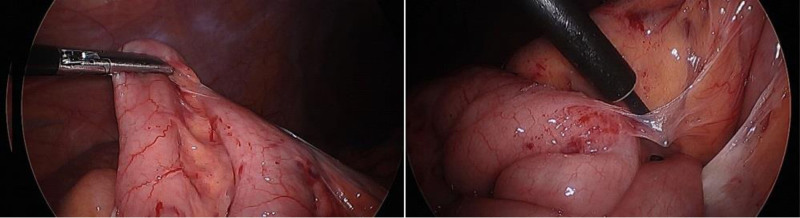
Strictured ileum with narrowed 17 cm “cocoon” of dense tissue concealing underlying perforation.

A previously healthy 14-year-old male presented with 1 year of worsening intermittent abdominal pain and acute constipation. He used no medications and endorsed no significant family or travel history. Initial physical examination revealed only mild generalized abdominal pain. Admission labs showed hemoglobin of 10.4 g/dL, with normal albumin of 4.0 g/dL, C-reactive protein, and erythrocyte sedimentation rate. Stool was fecal occult blood test (FOBT) positive with elevated calprotectin 240 μg/g. Computerized tomography of the abdomen/pelvis with intravenous contrast and subsequent magnetic resonance enterography revealed dilatation of a mid to distal ileal segment up to 10 cm wide, with mucosal hyperenhancement, surrounding inflammatory ascites, reactive mesenteric adenopathy, and a tiny outpouching indicative of diverticula or inflammatory process. Prominent mucosal folds seen in the dilated segment were considered by the radiologist to be suggestive of gastric heterotopia. To evaluate this, Meckel’s scan (Fig. 1) was performed and was negative. Infectious stool studies were negative.

Given his chronic nonspecific symptoms, anemia, positive FOBT, and imaging findings concerning for inflammatory process, IBD was suspected. Purified protein derivative placed for potential steroid use was negative. Esophagogastroduodenoscopy and colonoscopy were normal grossly and microscopically. IBD serologic, genetic and inflammation panel was negative. One month later, elective double balloon enteroscopy was performed and was notable only for a narrowed but nonobstructed area of bowel 10 cm from the ileocecal valve, dilated most significantly at the mid ileum. Biopsies taken from the proximal terminal ileum, distal terminal ileum, and mid ileum showed normal histology. Mid ileal biopsies showed focal acute inflammatory changes, but were normal elsewhere. The patient remained clinically stable throughout this procedure.

**FIGURE 2.**
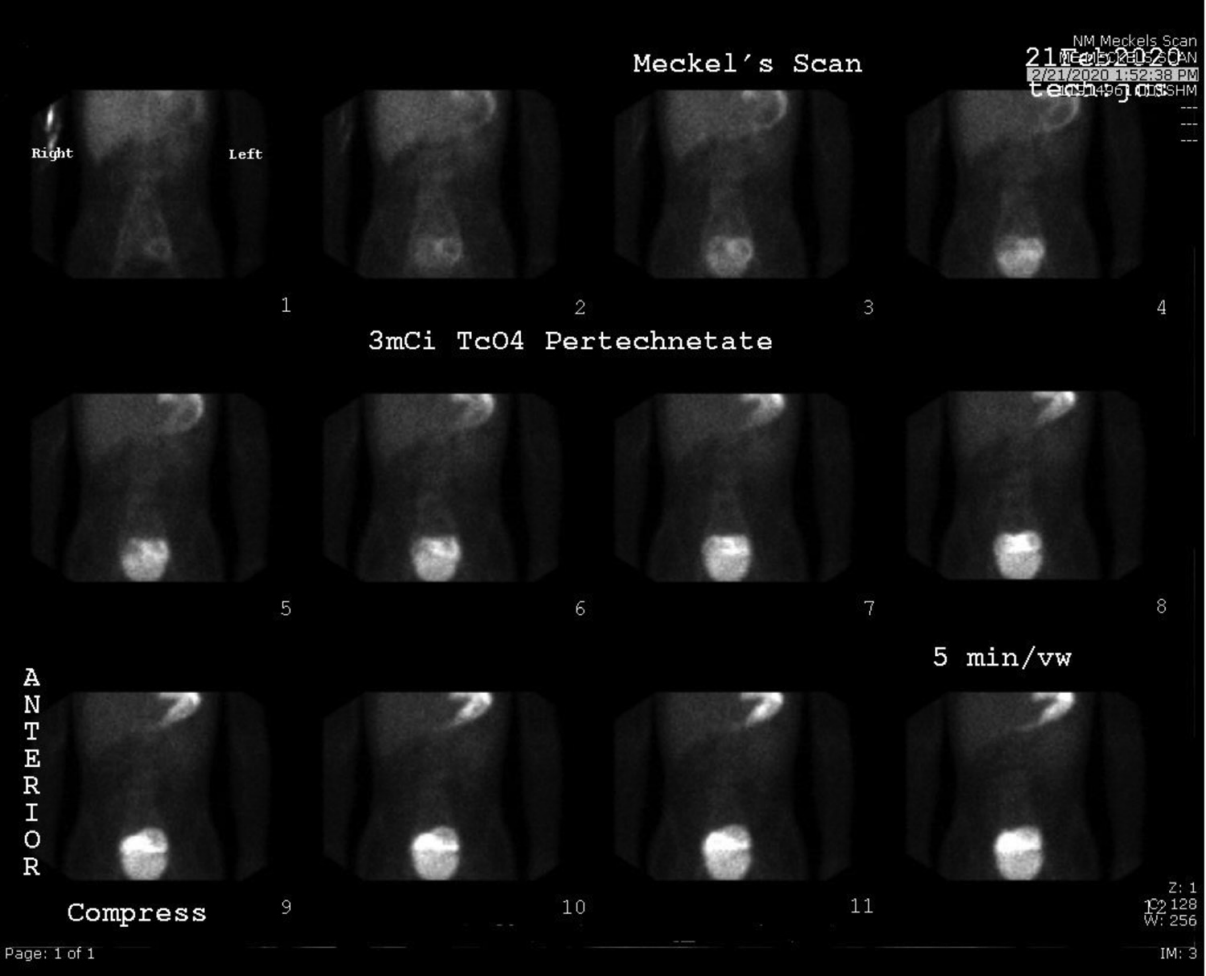
Negative Meckel’s scan with possible concealment of HGM activity by brightly contrasted bladder. HGM = heterotopic gastric mucosa.

**FIGURE 3.**
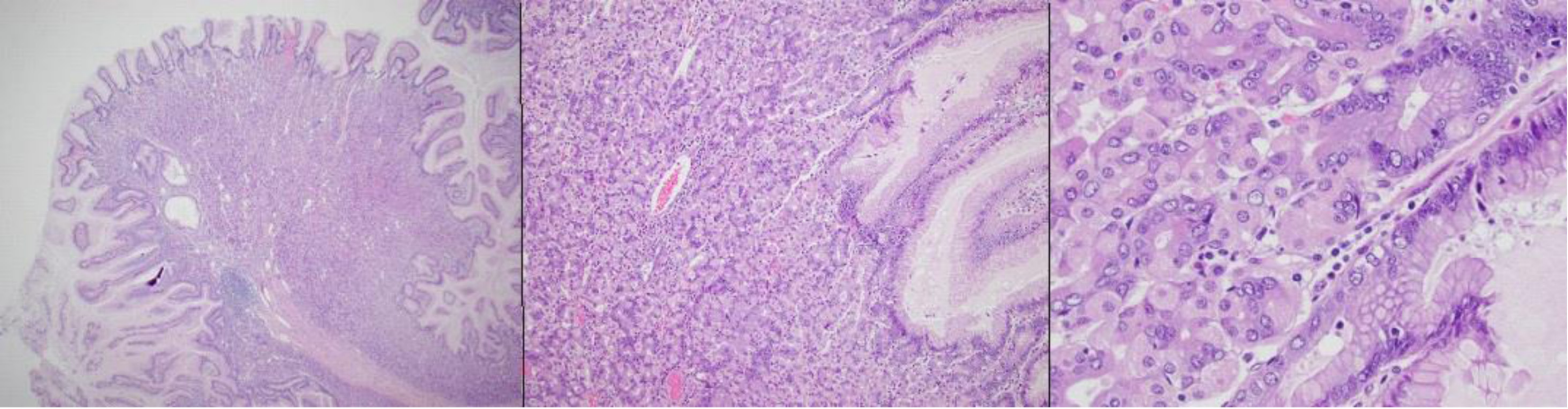
Low, high, and very high power resolution views of polypoid ectopic gastric oxyntic mucosa with serosal and subserosal fibrosis and adhesions.

The patient underwent exploratory laparoscopy that revealed a cocoon of dense intestinal adhesions proximal to the ileocecal valve, with multiple enlarged surrounding lymph nodes. After conversion to laparotomy and extensive lysis of adhesions, an inflammatory stenotic strictured area of ileum was found adhered to and concealing a perforation (Fig. 2). Seventeen centimeters of affected ileum was resected, and the remaining small bowel was reanastomosed. Specimen pathology showed 15 cm of polypoid ectopic gastric oxyntic mucosa, with serosal and subserosal fibrosis and adhesions (Fig. 3). The patient recovered quickly from surgery and was discharged 3 days postoperatively. At follow-up, he was well without recurrence of symptoms.

## DISCUSSION

This otherwise healthy male presented with chronic nonspecific symptoms of abdominal pain, mild anemia, and constipation. His labs were unremarkable with the notable exception of positive FOBT and moderately elevated fecal calprotectin and abnormal findings in the mid-ileum on computerized tomography and magnetic resonance enterography. These findings suggested the initial consideration of IBD. HGM was considered a potential etiology for the prominent folds found on imaging, but a negative Meckel’s scan contributed to our suspicion of IBD. When discussing the false-negative Meckel’s scan, we considered that the affected bowel segment may have been indistinguishable from the bladder due to close proximity (6, 7). This observation reinforces that the Meckel’s scan is neither conclusive nor reliable for the detection of HGM.

Endoscopy, colonoscopy, and double balloon enteroscopy were unable to distinguish the pathology in this case. HGM is generally reported to be grossly normal by endoscopic perception.^4^ Alternatively, capsule endoscopy has been found to result in a positive diagnosis rate ranging from 45% to 76%.^1^ In our patient, capsule endoscopy was not an option given our concern for ileal stricture inhibiting the passage of the capsule through the intestinal tract. Confocal laser endomicroscopy has been employed to identify HGM.^5^ Providing 1000 times magnification through the endoscope allows the user to differentiate normal from pathologic tissue. Future use of confocal laser endomicroscopy, under the right circumstances, could preclude the need for confirmatory biopsy. Narrow band imaging is an additional tool that allows visualization of the microvascular architecture of concerning tissue and can help differentiate ectopic pyloric-like mucosa and intestinal epithelium (5). As more studies emerge regarding the effectiveness of these techniques, they may become more widely available for use.

Surgical intervention is the most definitive approach for diagnosis and treatment of HGM (4). Endoscopic ultrasound and submucosal dissection have been reported to remove a lesion, as it was possible to detect dilated gastric glands and thickened mucosa (5). In that report, the lesion was large and only minimally involved the submucosal layer, thus making it amenable to endoscopic resection. It is important to remove the entirety of the involved lesion, as remaining ectopic gastric mucosa will continue to produce gastric acid, causing epithelial damage and predisposing to perforation and delayed or compromised healing of the reanastomosed segment (5). Following resection of HGM, patients generally have good outcomes.

HGM remains challenging to diagnose and treat due to the variable production of gastric acid in the tissue and spectrum of resultant symptoms ranging from abdominal pain and constipation to weight loss, anemia, and perforation (3). Labs can be unremarkable or misleading. Developments in endoscopic technology and techniques show promise for the future of diagnostic imaging for HGM and less invasive resection procedures. This patient provided a reminder of the uses and limits of laboratory testing and imaging modalities for intra-abdominal pathology, as well as the indications for surgical intervention. This case reinforces the importance of keeping a broad differential while evaluating nonspecific symptoms such as abdominal pain and unexplained bowel dilation, and that not all that strictures is IBD.

## ACKNOWLEDGMENTS

A.R. reviewed case, drafted manuscript, and approved final draft. T.R. reviewed case and approved final draft. M.K. reviewed case and approved final draft. A.N. reviewed case, assisted with figure creation, approved final draft. C.Y. reviewed case, revised manuscript, approved final draft. This paper has not been published or been considered for publication in any other journal at this time.
